# *PXDN*, *TCF4* and *TSPAN7* Are Differentially Expressed in B-Cell Acute Lymphoblastic Leukaemia: An Integrative Analysis

**DOI:** 10.3390/genes17060684

**Published:** 2026-06-10

**Authors:** Pasquale Primo, Francesco Cecere, Alessandra Cianflone, Fiorenza Mastrodonato, Giovanna Maisto, Luigi Coppola, Giovanna Giagnuolo, Fara Petruzziello, Luigi Vitagliano, Rosanna Parasole, Giuseppe Menna, Peppino Mirabelli

**Affiliations:** 1 UOS Laboratori di Ricerca e Biobanca, AORN Santobono-Pausilipon, Via Posillipo n.226, 80122 Naples, Italy; p.primo@santobonopausilipon.it (P.P.); f.cecere1@santobonopausilipon.it (F.C.); a.cianflone@santobonopausilipon.it (A.C.); f.mastrodonato@santobonopausilipon.it (F.M.); l.coppola@santobonopausilipon.it (L.C.); 2 UOC Oncoematologia Pediatrica, AORN Santobono-Pausilipon, Via Posillipo n.226, 80122 Naples, Italy; g.maisto@santobonopausilipon.it (G.M.); g.giagnuolo@santobonopausilipon.it (G.G.); f.petruzziello@santobonopausilipon.it (F.P.); r.parasole@santobonopausilipon.it (R.P.); g.menna@santobonopausilipon.it (G.M.); 3 Institute of Biostructures and Bioimaging, Consiglio Nazionale delle Ricerche, 80145 Naples, Italy; luigi.vitagliano@cnr.it

**Keywords:** paediatric leukaemia, dataset correlation, gene pathways

## Abstract

**Background/Objectives:** Acute lymphoblastic leukaemia (ALL) is a biologically heterogeneous disease in which transcriptional dysregulation contributes to disease onset and progression. Despite survival rates exceeding 90% in high-income countries, relapsed and high-risk cases remain a major clinical challenge, highlighting the need for improved molecular stratification, namely the classification of patients based on genetic and transcriptomic features associated with prognosis, therapeutic response, and disease biology, as well as for the identification of novel therapeutic targets. **Methods:** We performed an integrative cross-platform analysis to investigate the expression and potential relevance of three candidate genes: *PXDN*, *TCF4*, and *TSPAN7* in ALL. Gene expression was interrogated across the MILE microarray cohort and the St. Jude Cloud PeCan paediatric RNA-sequencing dataset. **Results:** Differential expression analyses consistently showed significant upregulation of *TCF4* and *PXDN* in B-cell ALL (B-ALL) across both platforms (adjusted *p* < 0.001), while *TSPAN7* displayed higher expression in T-cell ALL (T-ALL) and variable upregulation in B-ALL. These findings were supported by preliminary validation using quantitative PCR in paediatric B-ALL samples. To explore potential functional associations, we performed gene regulatory network inference using scGraphVerse, identifying differentially expressed genes putatively linked to *PXDN*, *TCF4*, and *TSPAN7*. Structural modelling using AlphaFold suggested candidate protein–protein interaction interfaces for a subset of these genes, although these predictions require experimental validation. Functional enrichment analysis indicated an over-representation of developmental pathways associated with *PXDN*- and *TCF4*-related networks, whereas *TSPAN7*-associated genes were enriched in processes linked to neuronal lineage development. **Conclusions:** Collectively, our results identify, for the first time, *PXDN*, *TCF4* and *TSPAN7* as differentially expressed genes in ALL and highlight the usefulness of integrative transcriptomic analyses across independent datasets. While limited by small-scale experimental validation and reliance on computational predictions, this study provides a framework for prioritising candidate genes and generates testable hypotheses regarding their potential involvement in leukaemia-associated molecular pathways.

## 1. Introduction

Acute lymphoblastic leukaemia (ALL) is a heterogeneous haematological malignancy arising from the clonal expansion of lymphoid progenitor cells, representing the most common cancer in children [1]. B-cell precursor ALL (B-ALL) represents approximately 25% of paediatric malignancies, with a peak incidence between 2 and 5 years of age, whereas T-cell ALL (T-ALL) typically presents later in childhood and adolescence [1,2]. Despite survival rates exceeding 90% in high-income countries, relapsed and high-risk cases remain a major clinical challenge, highlighting the need for improved biological stratification and novel therapeutic targets. Nevertheless, relapse and treatment resistance remain major causes of treatment failure, particularly in high-risk disease. Relapse and treatment resistance may arise through Darwinian clonal selection of resistant subclones under therapeutic pressure. The survival state can be guaranteed by the acquisition or persistence of genetic and epigenetic alterations, altered drug metabolism or apoptotic signalling, and protective interactions with the bone marrow microenvironment. In addition, quiescent leukaemia-initiating cells may evade cytotoxic therapies and later contribute to disease recurrence. In the immunotherapy setting, relapse may also involve antigen loss or modulation, lineage switch, impaired immune effector function, and immune escape. These mechanisms support the need for deeper transcriptomic characterisation to identify additional biomarkers and candidate pathways associated with disease persistence and recurrence [3]. Despite major therapeutic advances, treatment of paediatric ALL remains based on risk-adapted, multi-agent chemotherapy delivered through induction, consolidation/intensification, and maintenance phases, with treatment intensity increasingly guided by genetic features and minimal residual disease (MRD). In specific molecular subgroups, targeted agents have improved clinical management, including tyrosine kinase inhibitors for Philadelphia chromosome-positive or ABL-class-rearranged ALL. In recent years, immunotherapeutic approaches have further expanded the therapeutic landscape, particularly for B-ALL, including the CD19-directed bispecific T-cell engager blinatumomab, the anti-CD22 antibody–drug conjugate inotuzumab, ozogamicin, and CD19-directed CAR T-cell therapy in selected relapsed or refractory cases [4].

The molecular landscape of ALL is characterised by substantial genetic and epigenetic heterogeneity, including chromosomal rearrangements, copy number alterations, and transcriptional dysregulation [5,6,7,8]. Nevertheless, the heterogeneity of clonal lymphoid progenitor cells could generate a different therapy response and a different aggressiveness of the paediatric ALL [5]. Genomic mutations will lead to uncontrolled proliferation, impaired differentiation, and resistance to apoptosis. It is well known that B-ALL leukemogenesis involves a complex interplay of chromosomal translocation, copy number alterations, and mutations in key regulatory genes [6]. B-ALL recurrent genetic abnormalities, such as *ETV6-RUNX1*, *BCR-ABL1* (Philadelphia chromosome), *KMT2A* rearrangements, and *TCF3-PBX1*, have been well-characterised and are used for risk classification [7]. Also, transcriptomic studies have identified numerous overexpressed oncogenes (*MYC*, *BCL2*, *CRLF2*) and signalling pathways (*JAK-STAT*, *PI3K/AKT/mTOR*, *RAS/MAPK*) that contribute to leukaemia cell survival, proliferation, and drug resistance [8]. Recent advances in high-throughput sequencing and bioinformatics have enabled large-scale identification of differentially expressed genes (DEGs) in B-ALL [9]. Integrative analyses of datasets from RNA sequencing (RNA-seq), microarray studies, and single-cell transcriptomics have revealed novel candidate genes with potential roles in the leukemogenesis [10]. For example, overexpression of *CRLF2* is associated with *JAK-STAT* pathway activation and poor prognosis in high-risk B-ALL subtypes [11]. Similarly, *BCL2* upregulation promotes cell survival by inhibiting apoptosis, making it a target for venetoclax-based therapies. Additionally, *MYC* amplification drives metabolic reprogramming and aggressive disease phenotypes, highlighting its significance in relapse mechanisms [12]. While current diagnostic and prognostic frameworks rely on cytogenetics, immunophenotyping, and minimal residual disease assessment, increasing attention has been directed towards transcriptomic profiling to identify novel biomarkers and actionable pathways [9,10].

In this context, integrative analyses of publicly available datasets provide an opportunity to overcome the limitations of individual studies, such as restricted sample size, platform-specific biases, and limited availability of paediatric controls. Cross-platform validation, particularly between microarray and RNA-sequencing datasets, may enhance the robustness of candidate gene identification and prioritisation.

In the present study, we aimed to prioritise underexplored candidate genes emerging from cross-dataset transcriptomic analysis for subsequent experimental and computational evaluation. Gene selection was performed using a hypothesis-generating prioritisation strategy. Candidate genes were selected according to the following criteria: (i) overexpression in B-ALL in the MILE dataset; (ii) previous implication in oncological processes, including cellular differentiation, signalling pathways, tumour progression, or microenvironment-related mechanisms; and (iii) limited characterisation in paediatric B-ALL. On this basis, *PXDN*, *TCF4* and *TSPAN7* were selected for further investigation. *PXDN* was prioritised because it encodes a secreted peroxidase involved in extracellular matrix organisation and has been associated with tumour progression and immune modulation in several cancer types, in particular, melanoma-associated antigen MG50 [13]. *TCF4* was selected because of its role in transcriptional regulation, *Wnt*/β-catenin signalling, and cellular differentiation. Briefly, *TCF4* is a transcription factor acting downstream of the *Wnt/β-catenin* pathway, with established roles in cellular differentiation and oncogenic transcriptional programmes. It is a zinc-finger protein involved in the immunoglobulin expression and has been associated with epithelial-to-mesenchymal transition [14,15]. *TSPAN7*, also known as CD231, belongs to the tetraspanin family, a group of membrane proteins involved in signal transduction and cell–cell interactions and has been linked to tumour biology and haematological malignancies. *TSPAN7* was selected because, although previously linked to T-ALL and other oncological contexts, its expression pattern and potential relevance in B-ALL remain poorly defined [16,17].

Although these genes have been investigated in other oncological contexts, their expression patterns and potential functional relevance in ALL, particularly in paediatric disease, remain poorly defined. We therefore performed an integrative transcriptomic analysis combining the MILE microarray cohort and the St. Jude Cloud PeCan RNA-seq dataset to assess their differential expression across ALL subtypes.

To complement in silico findings, we conducted preliminary experimental validation using quantitative PCR in paediatric B-ALL samples. Furthermore, we explored putative gene regulatory networks and protein–protein interactions using scGraphVerse (version: Release 3.23) and AlphaFold(version 3.0)-based structural predictions, followed by functional enrichment analysis to identify associated biological processes.

Overall, this study aims to provide a preliminary framework for the identification and prioritisation of novel candidate genes in ALL through multi-dataset integration, while generating hypotheses regarding their potential involvement in disease-related molecular pathways.

## 2. Materials and Methods

### 2.1. MILE Study Dataset

The MILE study cohort comprises data from the MILE Stage I dataset, including 2143 retrospective adult and paediatric samples profiled using HG-U133 Plus 2.0 microarrays. Gene expression data were accessed through the BloodSpot platform (https://www.fobinf.com/, accessed on 10 March 2026), selecting the “MILE study” dataset within the leukaemia module. The expression levels of *PXDN*, *TCF4*, and *TSPAN7* were queried, and corresponding datasets were exported as .csv files for downstream analysis. Statistical comparisons between groups were performed using the Wilcoxon rank-sum test, with *p*-values adjusted for multiple testing using the Benjamini–Hochberg method.

### 2.2. St. Jude Cloud Dataset

Gene expression data were interrogated using the St. Jude Cloud PeCan “Visualization Community” tool (https://pecan.stjude.cloud/expression/gene-expression, accessed on 10 March 2026). For each gene of interest (*TSPAN7*, *TCF4*, and *PXDN*), the “Gene Expression Visualization” module was used to extract expression profiles across selected disease categories, including B-cell acute lymphoblastic leukaemia (B-ALL), T-cell acute lymphoblastic leukaemia (T-ALL), and myelodysplastic syndromes (MDS).

Expression data were visualised within the platform and subsequently exported as comma-separated (.csv) files, generating one dataset per gene. Each dataset included normalised expression values for individual samples, together with associated metadata such as sample identifiers, disease classification, and tissue type.

### 2.3. Study Population and Mononuclear Cells (BMMCs) Isolation

In this study, we included 11 BM samples from paediatric patients with childhood B-cell acute lymphoblastic leukaemia, classified as common B-ALL at diagnosis. The cohort comprised *n* = 8 males and *n* = 3 females, with an age range from 2 y/o to 16 y/o. Peripheral blood mononuclear cells were obtained from non-leukaemic paediatric subjects with normal blood counts.

After samples were withdrawn, whole blood EDTA was used for the purification of bone marrow mononuclear cells (BMMCs). The manual protocol for the density gradient separation of BMMCs began with the dispensation of 3 mL of HiSep™ LSM1077 (HiMedia Laboratories, Mumbai, India) in a conical sterile centrifuge tube. Then, peripheral blood was diluted 1:2 in Dulbecco’s phosphate-buffered saline (DPBS) solution w/o Ca^2+^ and Mg^2+^ (Sigma-Aldrich, St. Louis, MO, USA), stratified onto 3 mL of density gradient media and centrifuged at 400× *g* for 30 min and 4 °C. After centrifugation and using a sterile pipette, the BMMC layer was transferred to a new 15 mL centrifuge tube and diluted in DPBS supplemented with 2% foetal bovine serum (FBS). Two wash steps were performed before starting the controlled −1 °C/min freeze storage procedure or using the BMMCs for experimental procedures.

### 2.4. RNA Extraction and Retro-Transcription

Total RNA was extracted using the TRIzol™ reagent (Thermo Fisher Scientific, Waltham, MA, USA) following the manufacturer’s instructions. The starting material consisted of 2.0 × 10^5^ mononuclear cells obtained from an affected individual and 1 × 10^6^ peripheral blood mononuclear cells from non-leukaemia patients (without immunological or haematological pathologies). RNA concentration and purity were assessed using a NanoDrop™ spectrophotometer (Thermo Fisher Scientific, Waltham, MA, USA). For cDNA synthesis, 160 ng of total RNA was used as input. First-strand cDNA synthesis was carried out using the iScript™ cDNA Synthesis Kit (Bio-Rad, Hercules, CA, USA), according to the manufacturer’s protocol.

### 2.5. Quantitative PCR

Quantitative PCR was performed using 2 µL of cDNA per reaction. Reactions were set up with PrimePCR™ pre-validated primer assays for each gene and SsoAdvanced™ Universal SYBR^®^ Green Supermix (Bio-Rad, Hercules, CA, USA, https://www.bio-rad.com), following the manufacturer’s instructions. Quantitative PCR mix and amplification profile have been provided by the manufacturer. Amplification and detection were conducted on a Bio-Rad CFX real-time PCR system. Data were retrieved using CFX Maestro^®^ software (version 5.3.022.1030, Bio-Rad) and analysed using Microsoft Excel. Two technical replicates have been performed for each sample. Peripheral blood mononuclear cells (PBMCs) have been used as a reference control. Since we conducted the experiments on different plates, we normalised the qPCR analysis using the mean DC^t^*_B2M_* (*Beta-2-Microglobulin*).

### 2.6. Alphafold Analysis

Protein structure prediction and interaction modelling were performed using the AlphaFold Protein Structure Database (https://alphafoldserver.com/, accessed on 12 March 2026). The following proteins were analysed based on their UniProt accession numbers: TSPAN7 (P41732-1), PXDN (Q92626-1), TCF4 (P15884-1), NAV1 (Q8NEY1-1), LARGE1 (O95461-1), RGL1 (Q9NZL6-1), STK32B (Q9NY57-1), COL5A1 (P20908-1), DDR1 (Q08345-1), CORO2B (Q9UQ03-1), LRP6 (O75581-1), ZNF423 (Q2M1K9-1), CMTM8 (Q8IZU2-1), WFS1 (O76024-1), MYO5C (Q9NQX4-1), ZNF608 (Q9ULD9-1), EBF1 (Q9UH73-1), MYO1B (O43795-1), SHANK3 (Q9BYB0-1), TIAM2 (Q8IVF5-1), ECM1 (Q16610-1), TSPAN5 (P62079-1), EFNA1 (P20827-1), BCL7A (Q4VC05-1), SEMA6A (Q9H2F6-1), SMAGP (Q0VAI0-1), and BLK (P51451-1). Predicted protein structures were retrieved and used to explore potential protein–protein interactions among the selected candidates.

### 2.7. Network Analysis

Within the MILE dataset, all B-cell acute lymphoblastic leukaemia (B-ALL) samples were grouped and compared against non-leukaemia bone marrow controls to identify differentially expressed genes (DEGs). Differential expression analysis was performed using a linear modelling approach implemented in the limma package (v3.66.0) [18]. Genes were considered significantly differentially expressed if they met the following criteria: false discovery rate (FDR) < 0.001 and log2 fold change |(log2FC)| > 0.2. To investigate potential regulatory interactions involving *PXDN*, *TCF4*, and *TSPAN7*, we applied the scGraphVerse pipeline [19], inferring, comparing, and visualising gene networks (GNs) from expression profiles. The DEGs expression matrix was used as input for the *infer_networks()* function to obtain gene–gene interaction scores, setting GENIE3 as the method, keeping all the other parameters as default. Further, to obtain the final undirected weighted adjacency matrix, the scores data frame was first converted into a weighted adjacency matrix through the *adjacency_matrix()* function, then transformed into an undirected weighted adjacency matrix using the *symmetrise()* function. The top 10 gene interactors for *TSPAN7*, *TCF4* and *PXDN* were selected using the undirected weighted adjacency matrix, based on the interaction score. These genes with their predicted interactors were used for the functional enrichment analysis using g:Profiler (https://biit.cs.ut.ee/gprofiler/gost, accessed on 14 March 2026) to identify the overrepresented Gene Ontology (GO) biological terms.

## 3. Results

**Figure 1:**
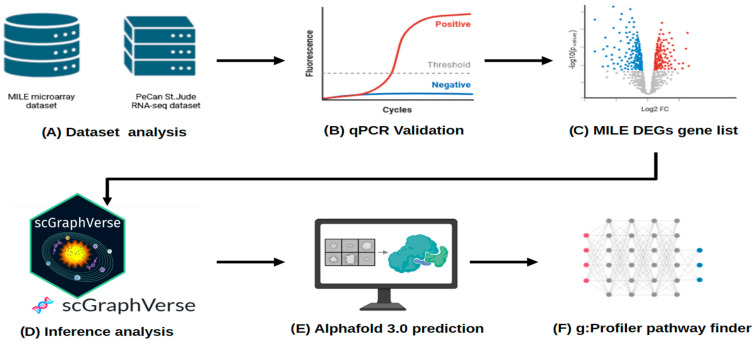
Study workflow for the integrated analysis of *TCF4*, *PXDN*, and *TSPAN7*. (**A**) Candidate genes were first interrogated across publicly available transcriptomic datasets, including the MILE microarray cohort and PeCan St. Jude platform, to assess differential expression patterns from both microarray and RNA-seq analysis. (**B**) Experimental validation was then performed by qPCR on leukaemia samples and normal bone marrow mononuclear cells, enabling a quantitative comparison of gene expression levels. (**C**) Top MILE DEGs were used as a query to find putative gene-to-gene interaction using the (**D**) scGraphVerse, followed by (**E**) the prediction of protein–protein interaction networks using AlphaFold 3.0. (**F**) Functional enrichment analysis was conducted using Gene Ontology (GO) profiling to identify associated biological processes.

In this study, the expression levels of *TCF4*, *PXDN*, and *TSPAN7* were interrogated across publicly available transcriptomic datasets, including the MILE study and the St. Jude Cloud PeCan platform. These findings were subsequently evaluated by quantitative PCR (qPCR) in leukaemia samples and compared with mononuclear cells isolated from non-leukaemia bone marrow aspirates, as summarised in the workflow (Figure 1).

To enable cross-dataset comparison, myelodysplastic syndrome (MDS) samples were used as a reference group in both the MILE and PeCan cohorts, representing the only common non-leukaemia comparator available across the two platforms for differential expression analysis.

### 3.1. MILE Cohort Analysis

**Figure 2:**
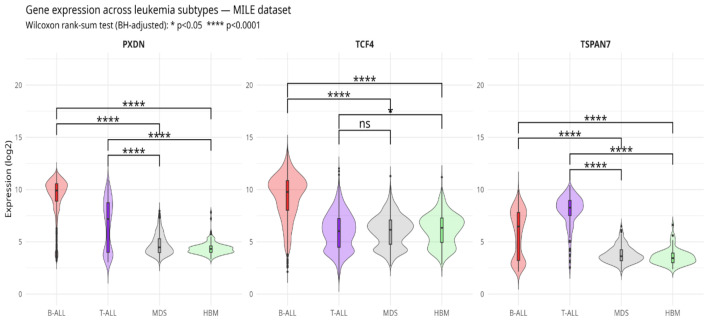
A violin-box plot representing the MILE study microarray analysis. The graphics colour code is red for B-ALL, violet for T-ALL, grey for MDS and green for healthy bone marrow (ns = not significant).

We consulted bloodspot.eu (https://www.fobinf.com/, accessed on 10 March 2026) and selected the MILE study (containing 2143 patients). We explored the expressions of *TSPAN7*, *TCF4*, and *PXDN* in both B-ALL and T-ALL compared to myelodysplastic syndromes (according to MILE study classification) and non-leukaemia bone marrow. We found a consistent over-expression in acute lymphoblastic leukaemia. Specifically, based on a Wilcoxon test, *PXDN* was overexpressed in both B-ALL and T-ALL (adjusted *p* < 0.0001) when compared with both MDS and healthy bone marrow (HBM); *TCF4* was significantly overexpressed in B-ALL (adjusted *p* < 0.001) but not in T-ALL (adjusted *p* > 0.05). Whilst *TSPAN7* was overexpressed in both B-ALL (adjusted *p* < 0.0001) and T-ALL (adjusted *p* < 0.0001) (Figure 2, Supplementary File S1).

### 3.2. St. Jude Cloud PeCan Analysis of Paediatric ALL

Following the identification of *PXDN*, *TCF4*, and *TSPAN7* deregulation in the MILE microarray dataset, we interrogated an independent RNA-sequencing cohort using the St. Jude Cloud PeCan platform (https://pecan.stjude.cloud/expression/gene-expression, accessed on 12 March 2026), which comprises paediatric leukaemia samples from both B-cell (B-ALL) and T-cell (T-ALL) acute lymphoblastic leukaemia. The use of RNA-seq data enabled a higher-resolution assessment of gene expression and provided an opportunity to validate microarray-derived findings in a paediatric-specific context.

**Figure 3:**
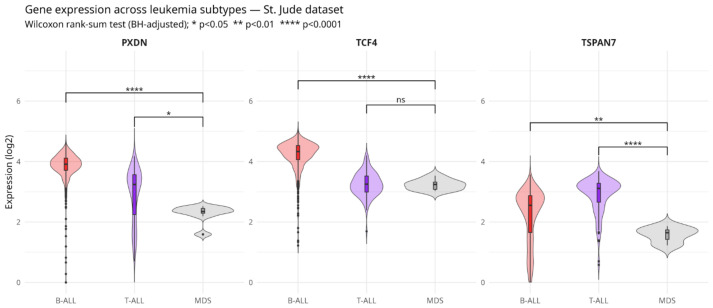
A violin-box plot representing PeCan st. Jude RNA-seq expression analysis comparison. The graphics colour code is red for B-ALL, violet for T-ALL, grey for MDS (ns = not significant).

Consistent with the MILE analysis, *TCF4* was significantly upregulated in B-ALL compared with MDS (adjusted *p* < 0.0001), while no significant difference was observed in T-ALL (adjusted *p* > 0.05), supporting a lineage-restricted expression pattern. *PXDN* showed significant upregulation in both paediatric B-ALL (adjusted *p* < 0.0001) and T-ALL (adjusted *p* < 0.05) relative to MDS, indicating a more broadly conserved transcriptional signature across ALL subtypes. *TSPAN7* was also significantly overexpressed in B-ALL (adjusted *p* < 0.01); notably, its expression was significantly higher in T-ALL compared to B-ALL (adjusted *p* < 0.001), reinforcing a preferential association with the T-lineage. Overall, these findings corroborate the cross-platform reproducibility of *PXDN* and *TCF4* upregulation in B-ALL and highlight subtype-specific expression patterns for *TSPAN7* (Figure 3, Supplementary File S2).

### 3.3. Validation Using Quantitative PCR (qPCR)

**Figure 4:**
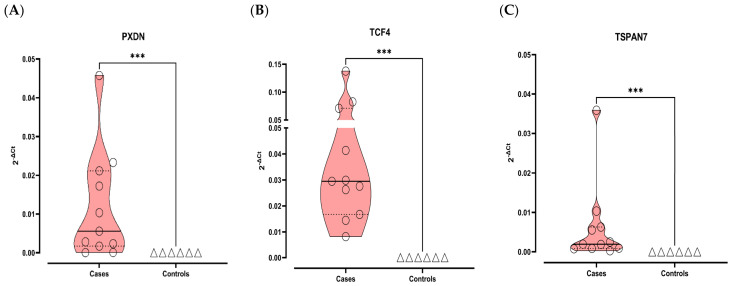
The violin plot shows the upregulation in terms of 2^−ΔCt^ of (**A**) *PXDN*, (**B**) *TCF4* and (**C**) *TSPAN7* in B-ALL BM samples when compared to PBMNCs from unaffected controls. In all cases, the median value of 2^−ΔCt^ was significantly higher (*** *p* < 0.001, unpaired Mann–Whitney U test) when compared to the control group. The continuous line in the violin plot shows the median value, and the upper and lower dotted lines show the 25th and 75th percentiles, respectively. The *B2M* gene has been used as a normalisation gene.

To provide preliminary experimental support for the transcriptomic findings, quantitative PCR (qPCR) was performed on 11 paediatric B-cell acute lymphoblastic leukaemia (B-ALL) samples collected at diagnosis and compared with five controls (non-leukaemia peripheral blood samples, i.e., no immunological nor haematological alteration had been identified). Quantitative RT-PCR results, expressed as 2^−ΔCt^, are displayed in Table 1 and plotted in Figure 4. In agreement with the *in silico* analyses performed on the MILE study and St. Jude PeCan datasets, qPCR confirmed a significant upregulation (*p* < 0.001, Unpaired Mann–Whitney U test) of *PXDN*, *TCF4*, and *TSPAN7* in B-ALL samples compared with controls (Supplementary File S3). *PXDN* showed higher expression levels in B-ALL cases, with a median value of 5.5 × 10^−3^ compared with 2.01 × 10^−5^ in controls. Similarly, *TCF4* was markedly increased in B-ALL samples, showing a median expression of 2.95 × 10^−2^ compared with 2.77 × 10^−3^ in controls. *TSPAN7* also displayed increased expression in B-ALL cases, with a median value of 1.9 × 10^−3^ compared with 3.86 × 10^−4^ in controls.

As shown by the violin plots, *PXDN* and *TCF4* exhibited a more consistent increase across B-ALL samples, although some degree of inter-patient variability was observed. By contrast, *TSPAN7* showed a more heterogeneous expression pattern, with most cases displaying low-to-moderate expression and a subset of samples showing markedly higher values. Overall, these findings support the transcriptomic evidence of deregulation of *PXDN*, *TCF4*, and *TSPAN7* in paediatric B-ALL and provide preliminary experimental validation of their increased expression in diagnostic B-ALL samples.

### 3.4. Gene Network Interaction

**Figure 5:**
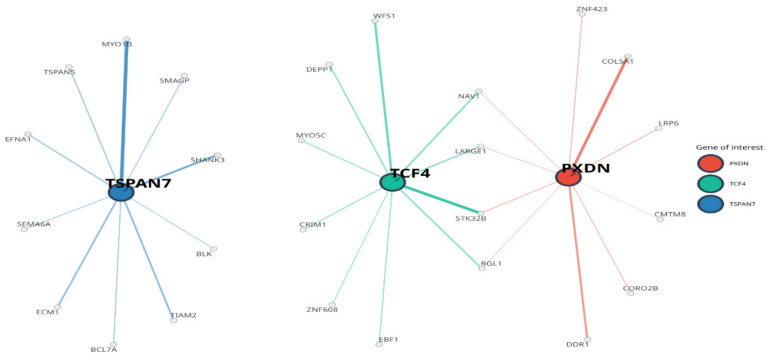
scGraphVerse result, relations between *TSPAN7* (blue), *PXDN* (red) and *TCF4* (green) with the top 10 DEGs. Smaller dots represent the top ten gene interactors. Line thickness represents the weighted regressed interaction score, whilst line length is not representative.

Since *PXDN*, *TSPAN7*, and *TCF4* seemed to be upregulated in B-ALL when compared with HBM and MDS, we sought to further explore their potential biological significance. We tried to identify deregulated genes that might be *TCF4*, *TSPAN7* and *PXDN*’s possible interactors. We used a tool for inferred analysis, scGraphVerse. The tool can identify if a group of genes can act in the same biological pathway. To identify a list of B-ALL-deregulated genes, we compared B-ALL samples with non-leukaemia bone marrow controls in the MILE dataset. We identified 802 unique differentially expressed genes (DEGs) (adjusted *p* < 0.001, |logFC| > 0.2, Table S1), both up- and downregulated, which were used as input for a network inference pipeline (scGraphVerse, https://www.bioconductor.org/packages/release/bioc/html/scGraphVerse.html, accessed on 12 March 2026). This analysis enabled the identification of putative interactors (among the 802 selected DEGs) for *TCF4*, *PXDN* and *TSPAN7* genes. We highlighted the top 10 interactor genes, reported in the image below (Figure 5, Table S2).

### 3.5. Predicted Structural Protein–Protein Interaction and Network Analysis

**Figure 6:**
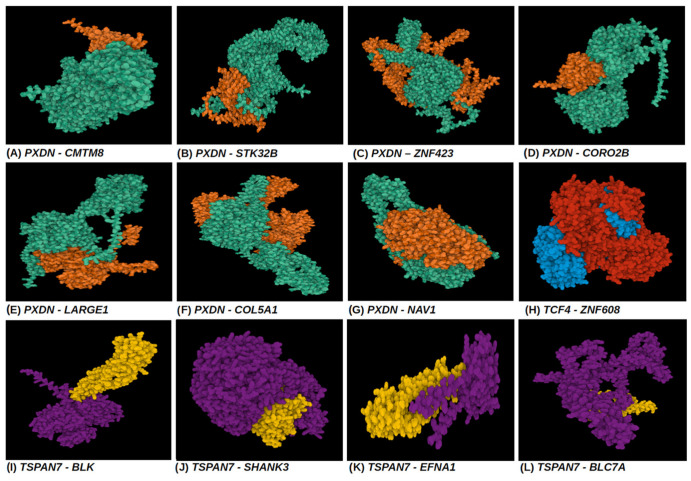
Alphafold 3.0 prediction of putative interactors of TCF4, TSPAN7 and PXDN previously found by scGraphVerse. We arbitrarily only represented interactions with a pLDDT > 50 spanning the most protein–protein complex from an x-axis and a y-axis. PXDN is in green, and its predicted possible interactors, (**A**) CMTM8, (**B**) STK32B, (**C**) ZNF423, (**D**) CORO2B, (**E**) LARGE1, (**F**) COL5A1 and (**G**) NAV1, are in orange. TCF4 is in red, and its predicted possible interactor, (**H**) ZNF608, is in blue. TSPAN7 is in yellow, and its predicted possible interactors, (**I**) BLK, (**J**) SHANK3, (**K**) EFNA1 and (**L**) BCL7A, are in violet.

To evaluate the scGraphVerse prediction, we tried to establish the interaction between TCF4, TSPAN7 and PXDN and their interaction by interrogating AlphaFold 3.0 (https://alphafoldserver.com/, accessed on 12 March 2026). We opt to employ Alphafold 3.0 since we sought to support the inferred analysis previously obtained. Alphafold software can predict the protein structure. Its upgrade, AlphaFold 3.0, also allows to identify possible protein-to-protein interactions based solely on their structure. We queried PXDN, TCF4 and TSPAN7 singularly with their putative interactors. We were able to predict the following interactions: PXDN-CMTM8, PXDN-STK32B, PXDN-ZNF423, PXDN-CORO2B, PXDN-LARGE1, PXDN-COL5A1 (as already known), PXDN-NAV1, TCF4-ZNF608 (the only interaction for TCF4 predicted), TSPAN7-BLK, TSPAN7-SHANK3, TSPAN7-EFNA1 and TSPAN7-BCL7A (Figure 6).

Finally, functional enrichment analysis performed on *TCF4, TSPAN7* and *PXDN* and their top 10 interactors (Table S2) using g: Profiler (https://alphafoldserver.com/welcome, accessed on 12 March 2026) suggested that genes associated with *TCF4* and *PXDN* were enriched in biological processes related to system development (GO:0048731). Conversely, *TSPAN7*-associated genes showed enrichment for terms linked to neuronal lineage development (GO:0048667 and GO:0048812). In the end, we used STRING (https://string-db.org/, accessed on 12 March 2026) and queried the most well-known gene responsible for B-ALL and T-ALL, along with our three genes. *TCF4* and *PXDN* seem to interact with other operators in both B-ALL and T-ALL pathogenesis, whilst *TSPAN7* does not seem to interact with any of the other genes (Supplementary File S4). Overall, these findings provide preliminary insights into potential shared and distinct transcriptional programmes associated with these genes, although further validation will be required to clarify their functional relevance in B-ALL.

## 4. Discussion

In this study, we employed an integrative multi-platform approach to investigate the expression and potential biological relevance of *PXDN*, *TCF4*, and *TSPAN7* in acute lymphoblastic leukaemia. By combining microarray data from the MILE cohort with RNA-sequencing data from the St. Jude Cloud PeCan dataset, we identified consistent transcriptional upregulation of these genes across independent datasets, thereby strengthening the robustness of our observations despite inherent differences in technological platforms and cohort composition.

Among the three genes analysed, *TCF4* and *PXDN* showed reproducible overexpression in B-ALL across both datasets and were further supported by preliminary qPCR validation. These findings suggest that these genes may be associated with transcriptional programmes active in leukaemia cells. *TCF4*, a key effector of the *Wnt*/β-catenin pathway, has been implicated in oncogenic transcriptional regulation and cellular plasticity in multiple tumour types. Also, considering that *Wnt*/β-catenin pathway alterations have already been found in ALL pathogenesis and other solid tumours [14,15,18], its upregulation in B-ALL may reflect activation of developmental pathways contributing to leukaemic cell survival or differentiation arrest. Similarly, *PXDN*, a secreted extracellular matrix-associated peroxidase, has been linked to tumour progression and microenvironment modulation in solid cancers [13,18]. Its consistent upregulation in ALL raises the possibility of a previously underexplored role in the leukaemia niche or extracellular signalling dynamics. *TSPAN7* displayed a more heterogeneous expression pattern, with higher expression observed in T-ALL compared to B-ALL, consistent with previous reports in haematological malignancies. Moreover, *TSPAN7* is also known as *T-ALLA*, and it is a target of *TAL1*, a gene responsible for T-ALL pathogenesis. The partial validation, i.e., *TSPAN7*, resulted in a milder overexpression, observed in our qPCR cohort, which may reflect biological variability or lineage-specific expression patterns. Given its role in membrane organisation and signal transduction, *TSPAN7* may contribute to subtype-specific signalling networks, although further investigation is required to clarify its functional significance in ALL since it has also been observed in glioma and T-ALL [16,19]. To gain insight into potential molecular interactions, we performed gene regulatory network inference using scGraphVerse, identifying a set of differentially expressed genes potentially associated with *PXDN*, *TCF4*, and *TSPAN7*. Subsequent structural predictions using AlphaFold suggested possible protein–protein interaction interfaces for a subset of these candidates. AlphaFold is a prediction tool used to analyse protein structure. Recently, AlphaFold 3.0 also allows us to predict multiple protein structures, thus it has been used to evaluate how the interaction found on scGraphVerse may be justified from a protein point of view. However, these findings should be interpreted with caution, as computational predictions do not constitute experimental evidence of direct interaction and require orthogonal validation. Nevertheless, to further corroborate our analysis, we queried the three candidate genes in STRING together with selected genes belonging to major ALL-related signalling axes, namely *PIK3CA*, *AKT1* and *MTOR* for the *PI3K*/*AKT*/*mTOR* pathway in B-ALL [20] and *NOTCH1* and *TCF7* for the *NOTCH1*-related T-ALL [21]. STRING was used as an exploratory tool to assess predicted protein–protein and functional associations, rather than as evidence of direct physical interactions [22].

In this analysis, *PXDN* showed predicted functional associations with *PIK3CA*, *AKT1* and *MTOR* (Supplementary File S4A). Although *PXDN* is not a canonical component of the *PI3K*/*AKT*/*mTOR* pathway, it encodes an extracellular matrix-associated peroxidase involved in collagen IV crosslinking and H_2_O_2_-dependent oxidative reactions [23]. Therefore, we hypothesised that *PXDN* may contribute indirectly to *PI3K*/*AKT*/*mTOR*-related signalling through extracellular matrix remodelling, redox homeostasis, and adhesion-dependent survival pathways. *TCF4* (Supplementary File S4B) showed a more restricted association, mainly with *AKT1*. Accordingly, *TCF4* should not be considered a canonical component of the *PI3K*/*AKT*/*mTOR* pathway, but rather a bHLH transcriptional regulator potentially linked to *AKT*-dependent transcriptional programmes [24]. In contrast, *TSPAN7* did not show predicted direct associations with the selected nodes of either the *PI3K*/*AKT*/*mTOR* or *NOTCH1*/*TCF7* pathways in our *STRING* analysis.

Regarding the *NOTCH1*-related pathway, *PXDN* showed a predicted functional association with *NOTCH1* (Supplementary File S4D). Although *PXDN* is not a canonical component of the notch pathway, this finding suggests a possible indirect functional link between *PXDN* and *NOTCH1*-related signalling, potentially mediated by extracellular matrix organisation, redox-dependent mechanisms, or cell–cell/cell–matrix communication. Regarding *TCF4*, we found a predicted functional association with *TCF7*, but not with *NOTCH1* (Supplementary File S4E). This association may be biologically relevant, as *TCF7* is a transcriptional regulator involved in T-cell differentiation and has been functionally associated with *NOTCH1*-driven transcriptional programmes [25]. Finally, *TSPAN7* does not seem to be involved in the *NOTCH1* pathways (see Supplementary File S4C,F).

Despite the intriguing results, we are aware that this study has several limitations. First, the use of myelodysplastic syndrome samples as a reference group in cross-platform comparisons represents a compromise due to the lack of normal paediatric bone marrow controls in the RNA-seq dataset and may introduce confounding effects. Second, experimental validation was performed on a limited number of samples, restricting the generalizability of the findings. Third, network inference and structural predictions are hypothesis-generating approaches that require further experimental validation. Fourth, several AlphaFold 3.0 structural prediction framework intrinsic limitations must be considered when evaluating the prediction results. Briefly, AlphaFold 3.0 operates effectively within an in silico *in-vacuum* environment. Consequently, it fails to model protein in a defined cell-localisation, i.e., the prediction of an interacting segment which resides within an inaccessible transmembrane domain or a sterically unavailable extracellular loop. Moreover, the algorithm does not consider the physiological compartmentalisation. It does not account for disparate sub-cellular localisations; for example, it may predict high-affinity interactions between a membrane protein and a nuclear transcriptional factor, a phenomenon that is biologically implausible in vivo. Nevertheless, the confirmation of these protein–protein interactions could open new avenues for drug discovery and development. For example, the *PXDN-COL*5*A1* interaction may play a crucial role in reshaping the leukaemogenic environment by reorganising the extracellular matrix. The inhibition of these interactions using mimetic peptides or gene silencing via targeted antisense RNA could contribute to overcoming therapeutic resistance and improving disease outcomes.

The X-linked localisation of *TSPAN7* raises the possibility of sex-related differences in gene dosage or regulation; however, given the exploratory nature of this observation, no definitive conclusions can be drawn, and dedicated studies will be necessary to address this hypothesis.

In conclusion, our integrative analysis identifies *PXDN*, *TCF4*, and *TSPAN7* as differentially expressed genes in ALL and highlights the value of combining publicly available datasets with preliminary experimental validation. While the functional roles of these genes in leukemogenesis remain to be elucidated, our findings provide a rationale for further investigation into larger, well-characterised cohorts and experimental models.

## Figures and Tables

**Table 1:** Statistics summary of the qPCR result plotted in Figure 4. Results are expressed as 2^−DCt^.

	*PXDN*		*TCF4*		*TSPAN7*	
	Cases (11)	Controls (5)	Cases (11)	Controls (5)	Cases (11)	Controls (5)
Minimum	5.0 × 10^−2^	1.3 × 10^−6^	8.2 × 10^−3^	4.57 × 10^−4^	2.0 × 10^−4^	2.29 × 10^−5^
25% Percentile	1.7 × 10^−3^	9.85 × 10^−6^	1.67 × 10^−2^	7.73 × 10^−4^	8.0 × 10^−4^	5.74 × 10^−5^
Median	5.5 × 10^−3^	2.01 × 10^−5^	2.95 × 10^−2^	2.77 × 10^−3^	1.9 × 10^−3^	3.86 × 10^−4^
75% Percentile	2.11 × 10^−2^	4.40 × 10^−5^	7.104 × 10^−2^	5.69 × 10^−3^	6.3 × 10^−3^	6.81 × 10^−4^
Maximum	4.57 × 10^−2^	6.27 × 10^−5^	1.378 × 10^−1^	6.96 × 10^−3^	3.58 × 10^−2^	7.81 × 10^−4^

## Data Availability

The MILE study cohort (https://www.fobinf.com/) and the St. Jude PeCan haematological malignancies dataset (https://pecan.stjude.cloud/expression/gene-expression). scGraphVerse (scGraphVerse https://www.bioconductor.org/packages/release/bioc/html/scGraphVerse.html), AlphaFold 3.0 (https://alphafoldserver.com/), Alexander Stand Database (https://scpca.alexslemonade.org/projects), G Profiler (https://biit.cs.ut.ee/gprofiler/gost).

## Supplementary Materials

The following supporting information can be downloaded at https://www.mdpi.com/article/10.3390/genes17060684/s1. Supplementary File S1, MILE study descriptive statistics; Supplementary File S2, PeCan St. Jude descriptive statistics; Supplementary File S3, *TCF4*, *TSPAN7* and *PXDN* qPCR results; Supplementary File S4, STRING results of TCF4, PXDN and TSPAN7 in *PI3K/AKT/mTOR* pathway and *NOTCH1* pathway; Table S1, top DEGs from MILE study; Table S2, top 10 interactors.

## Abbreviations

The following abbreviations are used in this manuscript:

ALL	Acute Lymphoblastic Leukaemia
MDS	Myelodysplastic Syndrome
PBMCs	Peripheral Blood Mononuclear Cells
GO	Gene Ontology
NGS	Next Generation Sequencing
MILE	Microarray Innovation in Leukaemia
DEG	Differentially Expressed Genes
T-ALL	T-cell Acute Lymphoblastic Leukaemia
B-ALL	B-cell Acute Lymphoblastic Leukaemia
BMMC	Bone Marrow Mononuclear Cell
HBM	Healthy Bone Marrow
BM	Bone Marrow
MRD	Minimal Residual Disease
B2M	Beta-2-Microglobulin
