# Supplementary figures and images for: PXDN, TCF4 and TSPAN7 Are Differentially Expressed in B-Cell Acute Lymphoblastic Leukaemia: An Integrative Analysis
Source: Genes (Basel). 2026 Jun 10;17(6):684. doi: 10.3390/genes17060684 (PMC13299483; doi:10.3390/genes17060684)

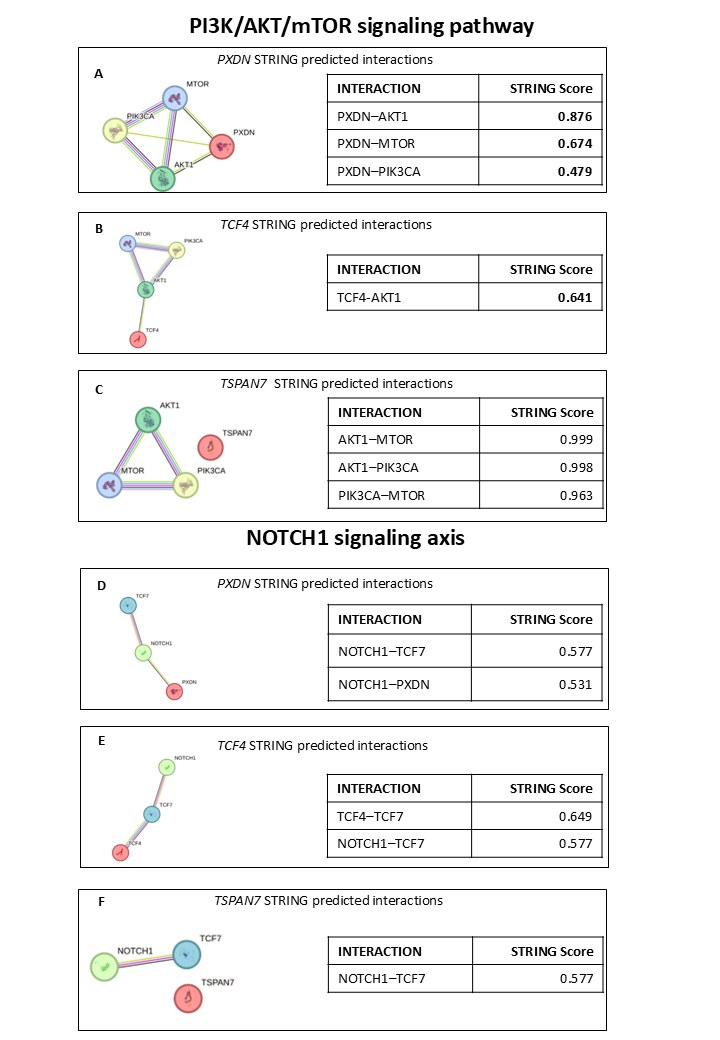

Supplement: Supplementary file 1 [file genes-17-00684-s001.zip › Supplementary file S4 - Pathway interactions_V1.png]
